# Apparent Homozygosity of p.Phe508del in *CFTR* due to a Large Gene Deletion of Exons 4–11

**DOI:** 10.1155/2014/613863

**Published:** 2014-02-06

**Authors:** Vassos Neocleous, Panayiotis K. Yiallouros, George A. Tanteles, Constantina Costi, Maria Moutafi, Phivos Ioannou, Philippos C. Patsalis, Carolina Sismani, Leonidas A. Phylactou

**Affiliations:** ^1^Department of Molecular Genetics, Function and Therapy, The Cyprus Institute of Neurology and Genetics, P.O. Box 23462, 1683 Nicosia, Cyprus; ^2^Department of Pediatrics, Hospital “Archbishop Makarios III”, 1474 Nicosia, Cyprus; ^3^Cyprus International Institute for Environmental & Public Health in Association with Harvard School of Public Health, Cyprus University of Technology, 95 Irenes Street, 3041 Limassol, Cyprus; ^4^Department of Clinical Genetics, The Cyprus Institute of Neurology and Genetics, P.O. Box 23462, 1683 Nicosia, Cyprus; ^5^Department of Cytogenetics and Genomics, The Cyprus Institute of Neurology and Genetics, P.O. Box 23462, 1683 Nicosia, Cyprus; ^6^Translational Genomics Team, The Cyprus Institute of Neurology and Genetics, P.O. Box 23462, 1683 Nicosia, Cyprus

## Abstract

We report a classic cystic fibrosis (CF) boy with a large deletion of exons 4–11 in the cystic fibrosis transmembrane conductance regulator (*CFTR*) gene on one allele and p.Phe508del in exon 10 on the second allele. Both parents of Georgian and Ukrainian background had no personal or family history of the disease. The initial molecular diagnostic investigation identified the patient as homozygous for the p.Phe508del and not compatible with his parent's genetic status. The possibility of nonpaternity or uniparental disomy (UPD7) was investigated and excluded using microsatellite analysis of highly polymorphic markers on chromosome 7. Array-CGH was also performed on the patient and revealed a male profile with a subtle deletion within the *CFTR* gene on the long arm (q-arm) of chromosome 7 (7q31.2). The deletion was confirmed by MLPA extending from probe L02380 to probe L14978 (28.7 kb) and that was inherited from his father, while p.PheF508del was inherited from his mother. These data highlight the need for additional testing for large deletions in patients with apparent homozygosity for a mutated *CFTR* allele that do not match the carrier status of the parents. Not testing can lead to misdiagnosis and misinterpretation of mutation carrier status and the expected penetrance of the disorder.

## 1. Introduction

Cystic fibrosis (CF) is an autosomal recessive disorder caused by mutations in the CF conductance transmembrane regulator (*CFTR*) gene [1, 2]. The *CFTR *gene spans an approximately 240 kb region on chromosome 7q31.3 and codes for 1480 amino acid protein that functions as a cAMP chloride channel in exocrine epithelia [3]. This channel regulates water and ion transport across membranes and is found in the epithelium of secretory epithelial cells in the lungs, liver, pancreas, intestine, reproductive tracts, and sweat glands. Mutations in the *CFTR* gene are responsible for both the classical and atypical presentations of the disease, including pulmonary disease, pancreatic insufficiency, malabsorption, meconium ileus, failure to thrive, infertility, and elevated concentrations of chloride in sweat [4, 5]. Currently, 1964 *CFTR* mutations have been listed in the CF database (http://www.genet.sickkids.on.ca/cftr/, accessed 3 February, 2014), 39 of which constitute 90% of mutations found in the Caucasian populations.

In patients with CF, the differentiation between true and apparent homozygosity for *CFTR* mutations is critical for correct prenatal diagnosis of CF, as well as for genetic counseling of the CF patient and his/her family members [6, 7]. Therefore, when routine molecular genetic analysis reveals apparent homozygosity for either rare or common *CFTR* mutations, it is important to confirm that this is a true homozygosity and not a false determination of homozygosity for a mutated *CFTR* allele. Homozygosity for common or rare mutations in the *CFTR* gene could be the result of a mutation on one allele and presence of a large deletion nearby the same sequence region on the second allele. In the present study, a patient with classic CF phenotype who has a large deletion of exons 4–11 of the *CFTR* gene on one allele and the classic p.Phe508del on the second allele is presented.

## 2. Case Report 

### 2.1. Clinical Evaluation and Molecular Analyses

The patient, a male infant now aged 16 months, is the first child of an unrelated couple of Georgian and Ukrainian origin. He was delivered by caesarean section at 36 weeks gestation because of polyhydramnios, ascites, and intestinal distention recovered on antenatal ultrasound scans. Soon after delivery he was transferred to the Neonatal Intensive Care Unit with abdominal distention and tenderness and on the same day he was taken to theatre for an exploratory laparotomy. Large quantities of meconium were recovered in the peritoneal cavity with many bowel adhesions and a volvulus of a segment of the small bowel that was ruptured at two points 15 cm apart. The bowel section between the two ruptures was severely ischaemic and was resected and two stomas were performed on the abdominal wall for the central and peripheral bowel colobomas. Molecular testing for *CFTR* gene [GENBank/Chromosome: 7; NC_000007.13 (117120017..117308719)] mutations with *ElucigeneCF29 V.2 kit* (Tepnel molecular diagnostics) and direct sequencing of exon 10 revealed homozygosity for p.Phe508del and the sweat test (diagnostic cut-off 60 mEqCl/Lt) performed at age one month yielded 100.3 mEqCl/Lt. The patient made a slow recovery from the peritonitis and, with introduction of feeds, demonstrated signs of severe malabsorption that were managed with an extensively hydrolyzed milk formula, pancreatic enzymes, and multivitamins supplements. He was sent home on day 19 of life but 18 days later he was re-admitted with severe dehydration, electrolyte imbalance, and metabolic acidosis. During this hospitalization, he developed septicaemia with enterobacter cloaca and subsequently an obstructive ileus due to a stricture of the central bowel coloboma that required a laparotomy and performance of a Bishop-Koop enterostomy. The enterostomy was closed successfully at age of four months and the patient is now growing satisfactorily along the 15th percentile for weight and 35th percentile for height on regular diet for his age and continuing pancreatic enzymes and multivitamins supplementation.

Following the confirmation of diagnosis, the carrier status of the parents was explored. Ethical review board approval and informed consent from both parents of the proband participating in the study was obtained in accordance with the national laws. The molecular diagnostic investigation of the parents revealed that the mother was heterozygous for the p.Phe508del whereas the father was not identified to have any of the tested genetic variations of the *CFTR* gene. Obviously, the above result was not compatible with the parent's genetic status and we expanded the investigations to exclude the possibility of nonpaternity. Chromosomal analysis performed only for the patient from peripheral blood samples by conventional G-banding techniques at the 550-band level revealed a normal karyotype and microsatellite analysis of highly polymorphic markers (D7S2212, D7S1808, D7S2201, D7S817, and D7S2204) on chromosome 7 also showed bi-parental inheritance. Thus, the presence of chromosome 7 uniparental disomy (UPD7) was excluded.

**Figure 1:**
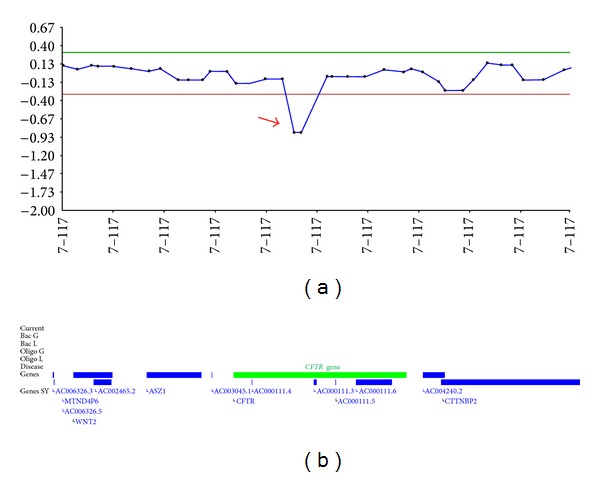
Array-CGH profile from the patient using Cytochip ISCA 180K Oligo platform (BlueGnome Ltd.) showing a subtle deletion (red arrow) of two oligonucleotides within the *CFTR* gene. Image from BlueFuse v3.2 (BlueGnome Ltd.) software.

Next, the possibility of compound heterozygosity of the proband bearing p.Phe508del on one allele inherited from the mother and a large deletion of the *CFTR* gene on the other allele inherited from the father encompassing the same sequence region including p.Phe508del was investigated by array-CGH. The array-CGH was carried out using the Cytochip ISCA Oligo array (BlueGnome Ltd.) with 180,000 oligos in a 4 × 180 k format according to the recommendations of the manufacturer and revealed on the patient a male profile with a subtle deletion within the *CFTR* gene on the long arm (q-arm) of chromosome 7 (7q31.2). The deletion included only 2 probes and the breakpoints of the deletion were found to lie between 117156104 and 117170728 and 117179672 and 117211736 (Genome Build GRCh37) (Figure 1).

**Figure 2:**
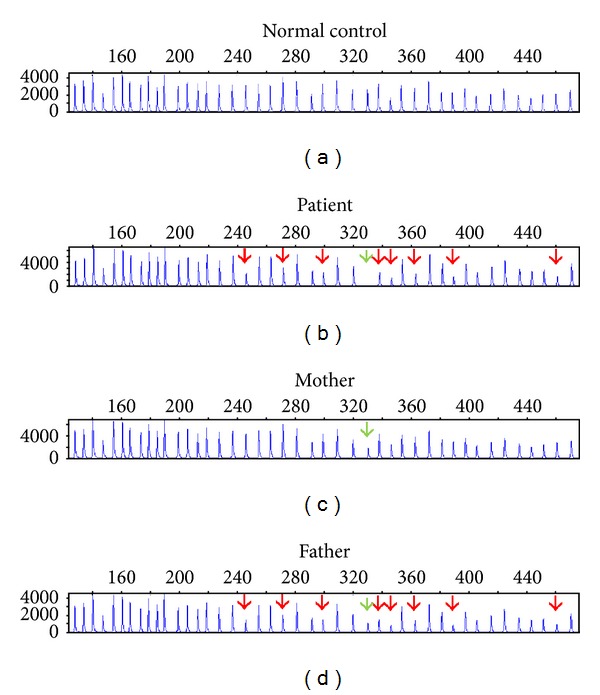
Multiple ligation probe amplification (MLPA) profiles using probe mixture mix P091-C1 CFTR (MRC-Holland). Red arrows showing the same deleted probes in the patient and his father in the *CFTR* gene. Nine probes are found to be deleted corresponding to a deletion size of 28.7 kb. Green arrows show the p.Phe508WT probe (*CFTR* probe 03322-L14978) which in the patient displayed no amplification due to the presence of the exonic deletion and the p.Phe508del mutation.

In order to confirm and further refine the breakpoints, multiplex ligation probe amplification (MLPA) with probe mix P091-C1 *CFTR* (MRC-Holland) was carried out (Figure 2). The MLPA probe mix contained probes for each of the 27 exons of the CFTR gene and nine probes were found to be deleted, extending from probe L02380 to probe L14978 (28.7 kb), that correspond to the respective deleted region found in the array-CGH. Therefore, the patient is a compound heterozygous for p.Phe508del mutation and a deletion of exons 4 to 11 (NCBI Exon Numbering); MLPA analyses of both parents revealed that the deletion was inherited from the father. MLPA analysis also confirmed the p.Phe508del mutation in the *CFTR* gene inherited from the mother.

## 3. Discussion

In this study, we reported a classic CF patient of mixed Georgian and Ukrainian ancestry carrying a large deletion spanning exons 4–11 in compound heterozygosity with the p.Phe508del. In the past, a similar large deletion spanning exons 4–10 was reported in a French female classical CF patient that also carried p.Phe508del on the other chromosome [8]. Meconium ileus is the earliest manifestation of CF occurring in up to 20% of the patients and is associated with certain *CFTR* mutations including p.Phe508del [9]. In some cases, bowel obstruction occurs antenatally presenting on ultrasound scans as dilated bowel, ascites, hyperechoic bowel, and calcifications [10]. Our patient had established small bowel volvulus and ruptures with peritonitis at birth and following a stormy course in the first days of life he went on to develop severe malabsorption and, at age of 37 days, an incident of severe dehydration with electrolyte imbalance which are typical manifestations for the CF population in Cyprus [11]. However, despite identification of two p.Phe508del in the patient, investigations of the carrier status in the parents gave inconclusive results on routine testing.

In general, mutation detection in the *CFTR* gene has mostly focused on point mutations, small deletions, and small insertions within the coding region of the gene. The actual frequency of large deletions may still be underestimated, because the majority of methods used for routine *CFTR* analysis are not suitable for detecting gross deletions or large rearrangements. In the past, large deletions and gross rearrangements in the *CFTR* gene would only be detected through the appearance of UPD inheritance and changes on Southern blots [12, 13]. With the recent development of modern techniques such as MLPA, array-CGH, and fluorescent multiplex PCR more genomic aberrations such as large deletions and gross rearrangements are a lot easier to identify [6, 7, 12, 14–16].

The case presented here unveils the limitations of the standard screening techniques used for the detection of mutations in the *CFTR* gene and denotes the importance of extending the genetic testing for CF. The observation that apparent homozygosity for p.Phe508del in the *CFTR* gene was caused by the presence of the large deletion spanning exons 4–11 confirmed our initial notion that the patient of the present study was a compound heterozygote for a large deletion. Up to date, the definite frequency of *CFTR* deletions is not yet fully known and varies depending on the population screened but is generally considered to account for less than 2% of CF chromosomes [17, 18]. Nevertheless, this value might increase if more cases of apparent homozygosity, like the one presented here, are resolved to be because of a large deletion or large rearrangement.

In conclusion, these data highlight the need of testing for large deletions in patients with apparent homozygosity for a mutated *CFTR* allele, since not testing can lead to misdiagnosis and to misinterpretation of mutation carrier status, expected penetrance, and its effects on protein function.

## References

[1] Rommens JM, Iannuzzi MC, Kerem B-S (1989). Identification of the cystic fibrosis gene: chromosome walking and jumping. *Science*.

[2] Collins FS (1992). Cystic fibrosis: molecular biology and therapeutic implications. *Science*.

[3] Bear CE, Li C, Kartner N (1992). Purification and functional reconstitution of the cystic fibrosis transmembrane conductance regulator (*CFTR*). *Cell*.

[4] Welsh MJ (1999). Gene transfer for cystic fibrosis. *Journal of Clinical Investigation*.

[5] Cohen TS, Prince A (2012). Cystic fibrosis: a mucosal immunodeficiency syndrome. *Nature Medicine*.

[6] Hantash FM, Rebuyon A, Peng M, Redman JB, Sun W, Strom CM (2009). Apparent homozygosity of a novel frame shift mutation in the *CFTR* gene because of a large deletion. *Journal of Molecular Diagnostics*.

[7] Diana A, Tesse R, Polizzi AM (2012). A large deletion causes apparent homozygosity for the D1152H mutation in the cystic fibrosis transmembrane regulator (*CFTR*) gene. *Gene*.

[8] Chevalier-Porst F, Bonardot A-M, Chazalette J-P, Mathieu M, Bozon D (1998). 40 kilobase deletion (CF 40 kb del 4-10) removes exons 4 to 10 of the cystic fibrosis transmembrane conductance regulator gene. *Human Mutation*.

[9] Carlyle BE, Borowitz DS, Glick PL (2012). A review of pathophysiology and management of fetuses and neonates with meconium ileus for the pediatric surgeon. *Journal of Pediatric Surgery*.

[10] Casaccia G, Trucchi A, Nahom A (2003). The impact of cystic fibrosis on neonatal intestinal obstruction: the need for prenatal/neonatal screening. *Pediatric Surgery International*.

[11] Yiallouros PK, Neocleous V, Zeniou M (2007). Cystic fibrosis mutational spectrum and genotypic/phenotypic features in Greek-Cypriots, with emphasis on dehydration as presenting symptom. *Clinical Genetics*.

[12] Schneider M, Joncourt F, Sanz J, Von Känel T, Gallati S (2006). Detection of exon deletions within an entire gene (*CFTR*) by relative quantification on the LightCycler. *Clinical Chemistry*.

[13] Tomaiuolo R, Sangiuolo F, Bombieri C (2008). Epidemiology and a novel procedure for large scale analysis of *CFTR* rearrangements in classic and atypical CF patients: a multicentric Italian study. *Journal of Cystic Fibrosis*.

[14] Hantash FM, Milunsky A, Wang Z (2006). A large deletion in the *CFTR* gene in CBAVD. *Genetics in Medicine*.

[15] Hantash FM, Redman JB, Starn K (2006). Novel and recurrent rearrangements in the *CFTR* gene: clinical and laboratory implications for cystic fibrosis screening. *Human Genetics*.

[16] Taulan M, Viart V, Theze C (2012). Identification of a novel duplication CFTRdup2 and functional impact of large rearrangements identified in the *CFTR* gene. *Gene*.

[17] Niel F, Martin J, Dastot-Le Moal F (2004). Rapid detection of *CFTR* gene rearrangements impacts on genetic counselling in cystic fibrosis. *Journal of medical genetics*.

[18] Paracchini V, Seia M, Coviello D (2008). Molecular and clinical features associated with *CFTR* gene rearrangements in Italian population: identification of a new duplication and recurrent deletions. *Clinical Genetics*.

